# Effects of extracorporeal shockwave therapy for mild knee osteoarthritis: A pilot study

**DOI:** 10.1097/MD.0000000000036117

**Published:** 2023-11-17

**Authors:** I Jun Choi, Jong Hu Jeon, Woo Hwa Choi, Hea-Eun Yang

**Affiliations:** a Department of Physical Medicine and Rehabilitation, Veterans Health Service Medical Center, Seoul, Republic of Korea.

**Keywords:** extracorporeal shockwave therapy, Kellgren–Lawrence grade, knee osteoarthritis, Lequesne index, ultrasound, visual analog scale

## Abstract

**Background:**

Extracorporeal shockwave therapy (ESWT) has been widely used for various musculoskeletal disorders, including knee osteoarthritis (OA), and has been shown in several studies to be a safe treatment. Although some studies have confirmed the pain-relieving effect of ESWT for knee OA, research on objectivity for structural changes in knee OA is lacking. The aim of this study was to evaluate the ESWT treatment mechanisms in patients with knee OA by means of clinical symptoms and ultrasound techniques as objective measures.

**Methods:**

Eighteen patients with mild knee OA were enrolled and randomized to 1 of 2 treatment groups: active or sham. Patients in the experimental group received 0.05 mJ/mm² total energy with 1000 pulses weekly for 3 weeks. We then assessed them before, immediately after, and 1-month after the last treatment using the following measurements: pain on a visual analog scale, Western Ontario and McMaster Universities Osteoarthritis Index, Lequesne index, knee joint range of motion, and ultrasonographic features (articular cartilage thickness, Doppler activity, and joint effusion height).

**Results:**

All 18 patients completed the 3 treatment sessions without any complication. Both the experimental and control groups improved in terms of OA symptoms, as measured by the visual analog scale, Western Ontario and McMaster Universities Osteoarthritis Index score, and Lequesne index (*P* < .05). The height of the suprapatellar effusion decreased with time course in the experimental group (*P* < .05) and showed significant differences with control group at 1-month follow-up (*P* < .05). The experimental group showed an increase in knee flexion range of motion and Doppler activity immediately following the last treatment session (*P* < .05), but the effect was not sustained at the 1-month follow-up.

**Conclusions:**

Although the therapeutic activity itself could improve OA symptoms, objective improvements were only observed after ESWT. Suprapatellar effusion height was reduced after ESWT and the effect was maintained after 1-month. Our results suggest that ESWT may be effective in reducing suprapatellar effusion and improving symptoms in mild knee OA. However, studies with a larger sample size are required.

## 1. Introduction

Knee osteoarthritis (OA) is one of the most common chronic degenerative joint disorders, resulting in arthritic symptoms such as joint pain, stiffness, limited range of motion (ROM), and functional loss.^[1]^

It is estimated that 10% of men and 13.0% of women over the age of 60 have symptomatic knee OA.^[2]^ Because knee OA eventually leads to physical disability and a disruption in quality of life, resulting in enormous social costs, early intervention for the treatment of knee OA is imperative.^[3]^

Medication and peri-articular injection of agents have traditionally been considered the first line of treatment for early knee OA such as Kellgren–Lawrence grade I or II. When conservative treatments fail in those patients with knee OA, they are frequently referred for surgical interventions such as arthroscopic debridement and total knee arthroplasty which results in more social costs.^[4]^ In this sense, extracorporeal shockwave therapy (ESWT) has emerged as an efficient and cost-effective treatment option for a variety of musculoskeletal disorders over the last 15 years, including lateral elbow epicondylitis, proximal plantar fasciitis, and calcifying shoulder tendonitis.^[5]^

In the process of applying ESWT to various musculoskeletal disorders, ESWT has been proposed as a treatment option for knee OA for several years. Zhao et al^[6]^ were the first to report the application of ESWT in knee OA in humans, demonstrating patient safety, pain relief, and functional recovery. To find the most appropriate anatomical region of knee OA for applying ESWT, in a preclinical study in rats, Wang et al^[7]^ found that ESWT treatment on the medial tibial subchondral bone produced better results than ESWT treatment on the lateral and other locations in the early OA knee. Additionally, Kim et al^[8]^ conducted a study on the dose-related effect of ESWT on knee OA and proposed the medium energy (0.093 mJ/mm^2^) treatment as the optimal intensity for knee OA. Recently, Avendaño-Coy et al^[9]^ reported a systematic review and meta-analysis of randomized clinical trials for ESWT on knee OA, proving pain and function improvements in patients. However, these studies only focused on the improvements in clinical symptoms which is subjected to the patient perception rather than objective improvements in radiologic features. To our knowledge, this is the first study to evaluate improvements in ultrasonographic features after ESWT on knee OA. The aim of this study is to demonstrate the effect of ESWT on mild knee OA not only in clinical symptoms but also in objective ultrasonographic improvements immediately following the treatment session, as well as the long-term effect of ESWT. By proving objective ultrasonographic improvements, ESWT would be more actively chosen as a first line therapy for mild knee OA.

## 2. Methods

### 2.1. Participants

We recruited patients with mild knee OA (Kellgren and Lawrence (K-L) grade 1–2) who were not indicated for surgery. Patients with K-L grade 1 to 2 were selected because they had a variety of treatment options and enough time to apply them. Patients with a K-L grade of 3 to 4 were excluded as they required more aggressive treatment options to improve their symptomatology. Patients with intact cognition (mini-mental state examination score > 20) and those who were ambulatory were included. Patients who had another musculoskeletal condition that could cause lower extremity pain, secondary causes of arthritis, or who had a knee intra-articular injection within the previous 6 months were excluded. Eighteen patients with knee OA were finally recruited for the study. Patients were randomly assigned to 1 of 2 groups: the experimental group (n = 9), which received ESWT, or the control group (n = 9), which received sham ESWT. All participants provided informed consent.

### 2.2. Intervention

ESWT and sham ESWT were administered by one physiatrist. A Dornier Aries system (Dornier MedTech Systems, GmbH, Germany) was used to deliver the shockwaves. Dornier Aries system uses flat electromagnetic shockwave emitter technique. In addition, it is composed of smart focus type providing a wide focal zone for surface and deep tissue treatment. Patients underwent three treatments at weekly intervals. For that, patients were positioned supine, with the treating knee flexed and one foot on the table.

At each treatment session, the experimental group received 1000 shockwaves at 0.05 mJ/mm^2^ on the affected knee’s proximal medial tibia. The control group received 1000 sham stimulation impulses at 0 mJ/mm^2^ on the same area. Patients and therapist could hear a sound similar to that of the regular ESWT, in order to enhance the sham design, but they were not able to see the dashboard.

### 2.3. Outcomes

Overall, two domains were evaluated: on the one hand, clinical symptoms such as pain, functional disability, and limitation of motion. On the other hand, objective measures, obtained by means of ultrasonography. Two researchers who were not involved in the randomization or intervention assessed all outcome measurements. Evaluations were conducted at 3 different points: at baseline, immediately after the completion of the third treatment session, and 1-month after the last treatment.

The visual analog scale (VAS), the Western Ontario McMaster University Osteoarthritis Index (WOMAC), the Lequesne index, and the knee joint ROM were used in order to assess symptomatology. The WOMAC, a validated disease-specific self-reporting questionnaire, was also used to assess OA symptoms.^[10]^ This WOMAC index consists of 24 items divided into 3 subscales that evaluate pain, stiffness, and function. The severity of symptoms was represented by a score ranging from 0 to 96 points, with a higher score representing worsening of symptoms. The Lequesne index was used to assess the disability of patients with knee OA. The Lequesne index contained 11 questions regarding knee discomfort, ambulation, and daily life difficulties.^[11]^ Its score ranged from 0 to 26 points, based on the severity of dysfunction, with a higher score indicating more dysfunction. A functional status was indicated with a score of <7 points.

For the ultrasonographic outcomes, we used the standardized musculoskeletal ultrasound scoring system for knee OA.^[12]^ This system enabled us to examine 14 anatomical sites and assess morphological changes, inflammation, and effusion, resulting in 61 items being scored.^[12]^ Since assessing 61 items was considered unnecessary, we opted to select 8 relevant items with presented more chances of change. Consequently, the suprapatellar recess was used to measure joint effusion height. Moreover, the medial joint space, medial parapatellar recess, the lateral joint space, and the lateral parapatellar recess were all tested in order to assess the Doppler activity. Finally, the thickness of the articular cartilage was measured at the medial trochlea, trochlear notch, and lateral trochlea.

### 2.4. Statistical analysis

Student *t* test and chi-square tests were performed to compare the baseline variables between the 2 groups. The treatment effect over time within each group and the difference in the treatment effect between the 2 groups were evaluated using repeated measures of ANOVA. An independent *t* test or Mann–Whitney *U* test were used for comparisons with the control group. A *P* value < .05 was considered statistically significant. Statistical Package for Social Sciences (SPSS) software (version 19.0) (SPSS Inc., Chicago, IL) was used for statistical analyses.

## 3. Results

All 18 patients completed the three treatment sessions without any complication. At baseline, no significant difference between experimental group and control group in terms of the characteristics and parameters were observed (Table 1).

**Table 1:** Demographics and baseline clinical characteristics.

	Experimental group (n = 9)	Control group (n = 9)	*P* value
Side (Rt:Lt)	4:5	5:4	
Age (yr)	73.7 ± 2.4	72.6 ± 2.3	.92
Weight (kg)	66.3 ± 8.9	72.7 ± 5.9	.23
Height (cm)	163.9 ± 5.0	165.7 ± 3.6	.39
BMI (kg/m^2^)	25.6 ± 2.9	26.4 ± 2.0	.46
K-L grade	1.3 ± 0.5	1.4 ± 0.5	.65
VAS	3.8 ± 2.1	3.7 ± 2.0	.91
WOMAC	31.9 ± 15.4	31.7 ± 15.2	.97
Lequesne index	9.5 ± 3.8	9.2 ± 3.1	.84

Values are presented as mean ± standard deviation.

BMI = body mass index, K-L grade = Kellgren–Lawrence grade, Lt = left, Rt = right, VAS = visual analogue scale, WOMAC = Western Ontario and McMaster University Osteoarthritis Index.

**Figure 1.**
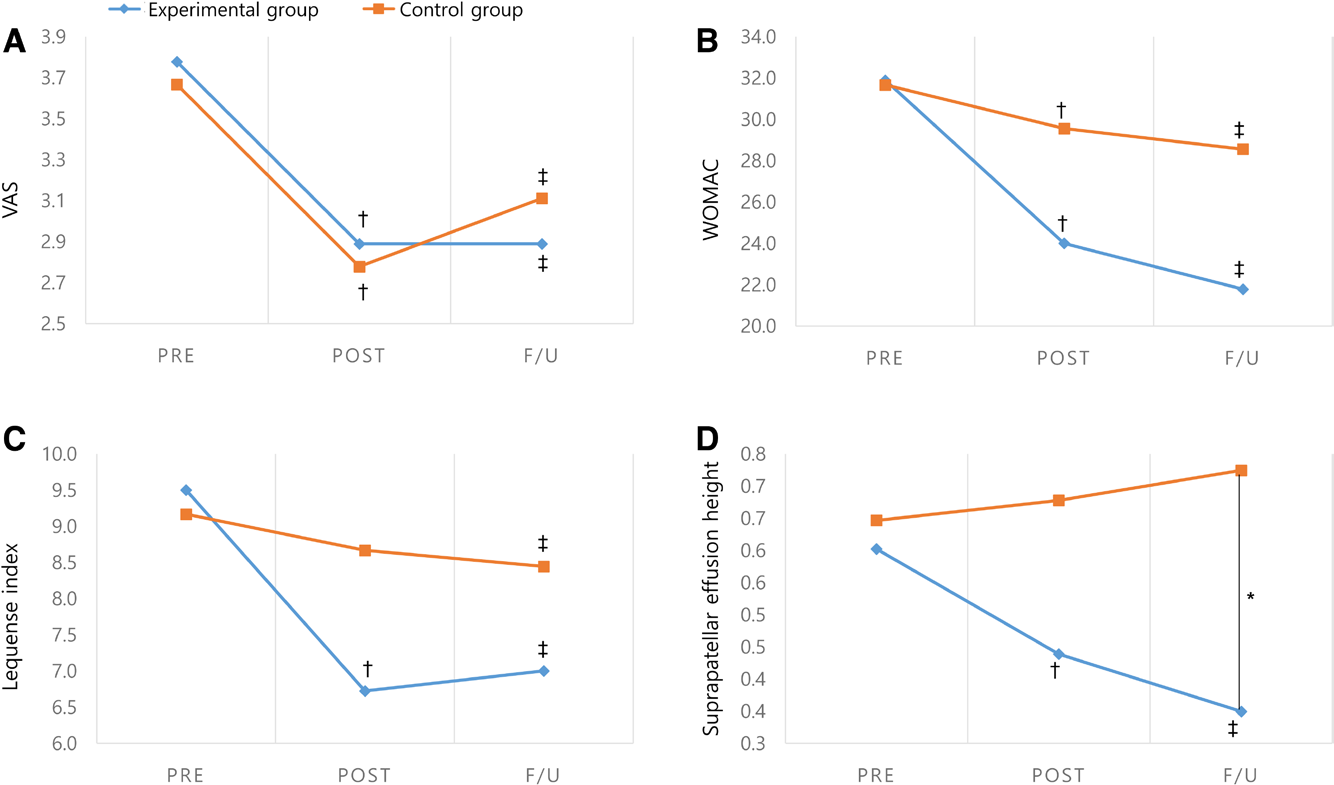
Treatment effect over time on VAS, WOMAC, Lequesne index, and suprapatellar effusion. VAS = visual analogue scale, WOMAC = Western Ontario and McMaster University Osteoarthritis Index.

Both experimental and control group improved their OA symptoms as measured by the VAS, WOMAC score, and Lequesne index (Fig. 1A–C). At the baseline, the VAS score in experimental and control group were 3.8 ± 2.1 and 3.7 ± 2.0, respectively. Following the treatment session, the VAS scores in each group were 2.9 ± 1.8 and 2.8 ± 1.6, respectively. At 1-month follow-up after the treatment, they were 2.9 ± 1.5 and 3.1 ± 2.1, respectively. Furthermore, the VAS score also increased in the control group after 1-month, but it was not statistically significant. At baseline, the WOMAC scores in each group were 31.9 ± 15.4 and 31.7 ± 15.2, respectively. After treatment, they were 24.0 ± 10.5 and 29.6 ± 14.7 and, at after 1-month follow-up, 21.8 ± 11.5 and 28.6 ± 14.0 in each group, respectively. WOMAC score improvement seemed to be greater in the experimental group, but intergroup differences were not statistically significant. At baseline, the Lequesne index was 9.5 ± 3.8 and 9.2 ± 3.1 in each group, respectively. After the treatment session, the Lequesne index was 6.7 ± 2.7 and 8.6 ± 3.1, respectively, and 7.0 ± 3.4 and 8.4 ± 3.3 after the 1-month follow-up for each respective group. Thus, the Lequesne index was improved over the entire period in the experimental group. In the control group, this improvement was uniquely observed at 1-month follow-up.

At 1-month follow-up, the suprapatellar effusion in the experimental group decreased over time and showed a significant difference from the control group (Fig. 1D). The height of the suprapatellar effusion was 0.6 ± 0.3, 0.4 ± 0.2, and 0.3 ± 0.2 at baseline, after treatment, and at 1-month follow-up, respectively, in the experimental group and were 0.6 ± 0.3, 0.7 ± 0.3, and 0.7 ± 0.2 in the control group.

VAS, WOMAC, and Lequesne index were improved in both experimental and control group. On the other hand, suprapatellar effusion decreased with time course only in experimental group.

The experimental group experienced an increase in knee flexion ROM and Doppler activity immediately following the last treatment session in experimental group, but the effect was not sustained at 1-month follow-up (Table 2).

**Table 2:** Increased parameters after the treatment in the experimental group.

	Pretreatment	Posttreatment	1 mon f/u	*P* value
Knee flexion ROM	112.8 ± 14.2	120.6 ± 9.5	114.4 ± 8.8	.49
DA medial	0.00 ± 0.00	0.67 ± 0.50	0.11 ± 0.33	.04
DA med para	0.00 ± 0.00	0.47 ± 0.52	0.35 ± 0.50	.35
DA lateral	0.00 ± 0.00	0.44 ± 0.52	0.33 ± 0.50	.35

Values are presented as mean ± standard deviation. *P* value provides the statistical significance for pretreatment and posttreatment difference.

DA lateral = Doppler activity at lateral joint space, DA med para = Doppler activity at medial parapatellar recess, DA medial = Doppler activity at medial joint space, ROM = range of motion.

Other parameters such as knee extension ROM, Doppler activity at lateral parapatellar recess, cartilage thickness at medial trochlea, trochlear notch, and lateral trochlea showed no time-dependent changes or intergroup differences.

## 4. Discussion

As previously stated in the background section, ESWT appears to be a promising alternative option among various treatment options for early mild knee OA. When compared to other treatments, ESWT presents several advantages, including noninvasiveness, a low complication rate, no hospitalization requirement, and a low cost. Although the mechanism of ESWT on knee OA is not completely understood, several preclinical studies have previously demonstrated the ESWT mechanisms on knee OA.^[1,13,14]^ In this sense, cartilage destruction and uncoupled subchondral bone remodeling are known to be critical degenerative changes in OA.^[15,16]^

ESWT acts as a compressive force during the positive phase and then as a tensile force and shear stress during the negative phase, causing microbubbles in liquid molecules and cavitational effects on the treatment area. This complex etiologic forces may lead to biophysical effects on target tissues.^[17]^ Wang et al applied ESWT on OA in a preclinical rat model, demonstrating a significant reduction in articular cartilage degradation via safranin-O staining, Mankin scores, matrix metalloproteinase 13, and levels of C-telopeptide of type II collagen, all of which contributed to chondroprotective effects in OA.^[7,18,19]^ Furthermore, ESWT has been shown to restore the production of IL-10 and TNF-α in chondrocytes to normal levels.^[20]^ ESWT has also been shown to alter von Willebrand factor, endothelial nitric oxide synthase, vessel endothelial growth factor (VEGF), morphogenetic protein 2 (BMP-2), alkaline phosphatase, and osteocalcin, all of which promote neovascularization and osteogenesis for subchondral bone remodeling.^[21]^ Ochiai et al^[22]^ demonstrated, by means of a histomorphologic analysis of tissues from ESWT-treated rats, a reduction in calcitonin gene-related peptide in the dorsal root ganglia neurons, which are closely related to joint pain sensation, suggesting a mechanism for symptom improvement for ESWT on knee OA.

Based on the revealing of ESWT mechanisms, several studies^[6,23–29]^ have demonstrated the safety and efficacy of ESWT on human knee OA. However, these studies are typically focusing on changes in clinical symptoms, with only 1 study on structural changes of knee OA using magnetic resonance imaging to evaluate bone marrow edema change.^[30]^ In addition, the morphological and hemodynamic changes in the knee joints were not included in the above-mentioned studies. As a result, we sought to assess the structural and morphological changes in knee OA with ESWT treatment.

Regarding the intensity of ESWT on the knee, previous research concluded that high intensity energy therapy may be beneficial, requiring only a single treatment and producing superior results.^[31]^ On the other hand, high-energy therapy may cause more pain, local swelling, tenderness, and a decrease in patient compliance, which may be considered inefficient.^[31,32]^ On molecular and ultrastructural levels, a single bout of ESWT (150 shockwaves of 0.5 mJ/mm^2^) caused degenerative changes in the hyaline cartilage of adult rats.^[33]^ Moreover, Kim et al^[8]^ compared low-energy therapy and medium-energy therapy in 2 groups, and the latter improved in a higher rate of their clinical symptoms. However, no histological or molecular safety assessments were performed in that study. In an in vitro study on human chondrocytes, cytotoxicity was measured at <10% with a dose of <0.06 mJ/mm^2^.^[34]^ Based on the results of our study, we determined that a dose of <0.06 mJ/mm^2^ was safe. Furthermore, a human study examining the effect of different levels of energy revealed that ESWT energy >0.04 mJ/mm^2^ was more effective.^[33]^ In light of these 2 studies, we applied an ESWT dose of 0.05 mJ/mm^2^ in our study. Furthermore, the latter human study^[33]^ found that 1000 ESWT shockwaves had no side effects or complications, so we applied the same shockwaves times in our study. Despite the fact that this was an in vitro study, we considered appropriate to choose an energy density of 0.05 mJ/mm^2^ based on a previous study of human chondrocytes.

As different targets were chosen in other studies,^[6,8,24,26,27,29]^ we chose the subchondral bone of the medial proximal tibia, as evidenced by our previous study,^[24]^ for the evidence that the subchondral bone area is critical for degeneration of knee OA.^[15,16]^

In our study, at the posttreatment evaluation, both experimental and control group improved in clinical symptoms related to knee OA as measured by the VAS, WOMAC score, and Lequesne index. In contrast to our findings, Uysal et al^[35]^ reported that radial ESWT showed statistically significant improvement in parameters such as VAS score, knee ROM, 20 meters walk test, and Lequesne index when compared to sham ESWT. Zhong et al^[29]^ compared ESWT to placebo treatment for knee OA, and the VAS, WOMAC, and Lequesne index were superior in the ESWT group in long-term follow-up. Although both groups in our study improved immediately after the last treatment session, at 1-month follow-up evaluation, the 3 measures showed better outcomes in experimental group compared to control group. Even though the intergroup differences between experimental and control groups were not statistically significant, due to Zhong et al^[29]^ have previously proved long-term effect of ESWT on clinical symptoms, we thus assumed that long-term follow-up is required to be consistent with previous studies.

Avendaño-Coy et al^[9]^ demonstrated greater ROM improvements in patients treated with ESWT compared to the control group in a systematic review and meta-analysis of randomized clinical trials related to ESWT on human knee. In the same vein, our study also found that knee flexion ROM improved after the final treatment session. This finding was consistent with those from Arno et al who reported that ESWT improved function by increasing perfusion in ischemic tissues, stimulating growth factors, decreasing inflammation, and accelerating healing.^[36]^

According to our findings, Doppler activity increased at medial joint space immediately following treatment. ESWT can lead to biologic response in target tissue by inducing anti-inflammatory effects, cell proliferation, and, in particular, neovascularization, which may result in tissue regeneration and repair.^[37]^ In a preclinical study in rats, Wang et al investigated the effects of ESWT on chondroprotection in knee OA of these animals, finding that the effects of ESWT on subchondral bone remodeling were supported by increased vascularization manifested by changes of von Willebrand factor, endothelial nitric oxide synthase, and VEGF.^[19]^ Especially, VEGF is not only a protein that promotes vasculogenesis and angiogenesis, but it is also a marker of increased vascular permeability and microvascular activity, including the angiogenic growth of new vessels.^[19]^ Our previous study also suggested a similar increase in Doppler activity after ESWT treatment, supporting our hypothesis that ESWT has a neovascularization effect on human knee OA.^[24]^

While other knee ROM and Doppler activity at other areas showed no change after treatment, suprapatellar effusion height decreased with time in the experimental group and showed a significant difference with the control group at 1-month follow-up. As previously stated, ESWT can induce a biologic response in target tissue by inducing anti-inflammatory processes.^[37]^ Moreover, ESWT was proved to reduce the level of C-telopeptide of type II collagen and matrix metalloproteinases in preclinical rat models with knee OA.^[19,38]^ By increasing the production of IL-10 and TNF-α in chondrocytes to normal levels, ESWT may also exert chondroprotective effects in OA models.^[20]^ Changes in cytochrome-c and cleaved caspase-3 levels relative to procaspase-3 were also decreased by ESWT, which showed a significant decrease in pro-inflammatory and cartilage degradation markers.^[39]^ Although these biological responses observed in animal studies have not been adequately demonstrated in ESWT treatment in human knee OA, we considered that these anti-inflammatory responses may help to reduce effusion from knee OA, eventually leading to a decrease in suprapatellar effusion height measured by ultrasound in our study.

To conclude, some limitations of this study should be noted. First, small sample size might have affected the results of this study. However, future studies with larger groups of subjects, based on the result of this pilot study, are warranted. Second, only 1 treatment protocol was selected in our study. There has been several studies^[6,23–29,39–45]^ on the ESWT on human knee OA, in which at least 14 different ESWT treatment protocols were chosen. Therefore, more investigation is required in order to determine the optimal protocol of ESWT on knee OA, considering dose, frequency, intensity, and location.

## 5. Conclusions

To sum up, this pilot study investigated the effects of ESWT on pain, function, and ultrasonographic features in patients with mild knee OA. This study revealed that ESWT was not only effective in reducing pain and improving knee function in patients with mild knee OA, but it also reduced suprapatellar effusion height immediately after treatment and even after a month of follow-up. Doppler activity increased after ESWT, but did not persist at 1-month. Because objective structural changes such as increased Doppler activity and decreased suprapatellar effusion height were observed, and especially because the decrease in suprapatellar effusion height lasted for a month, we can strongly recommend ESWT as a compromising treatment option for human knee OA. Despite the encouraging results, further investigations with larger study samples are necessary in order to determine the optimal regimen of ESWT on human knee OA.

## Acknowledgments

We would like to acknowledge and thank VHS Medical Center Research Grant.

## Author contributions

**Data curation:** Woo Hwa Choi.

**Formal analysis:** Woo Hwa Choi.

**Funding acquisition:** Hea-Eun Yang.

**Investigation:** I Jun Choi, Woo Hwa Choi.

**Methodology:** Jong Hu Jeon.

**Project administration:** Hea-Eun Yang.

**Resources:** Jong Hu Jeon.

**Software:** Jong Hu Jeon.

**Supervision:** Hea-Eun Yang.

**Writing – original draft:** I Jun Choi.

**Writing – review & editing:** Hea-Eun Yang.
